# Molecular diagnosis of infective endocarditis from culture-negative valve samples in a tertiary hospital in Iran

**DOI:** 10.1128/spectrum.01856-24

**Published:** 2025-01-31

**Authors:** Mohammad Reza Mohammadi, Ashraf Mohabbati Mobarez, Mohammad Ali Broumand, Neda Baseri, Mina Latifian, Saber Esmaeili

**Affiliations:** 1 Department of Bacteriology, Faculty of Medical Sciences, Tarbiat Modares University, Tehran, Iran; 2 Department of Pathology and Laboratory Medicine, Tehran Heart Center Tehran, University of Medical Sciences, Tehran, Iran; 3 Tehran Heart Center, Cardiovascular Diseases Research Institute, Tehran University of Medical Sciences, Tehran, Iran; 4 National Reference Laboratory for Plague, Tularemia and Q fever, Research Centre for Emerging and Reemerging Infectious Diseases, Pasteur Institute of Iran, Akanlu, Kabudar Ahang, Hamadan, Iran; 5 Department of Epidemiology and Biostatics, Research Centre for Emerging and Reemerging Infectious Diseases, Pasteur Institute of Iran, Tehran, Iran; Foundation for Innovative New Diagnostics, Geneva, Switzerland

**Keywords:** endocarditis, Iran, *Tropheryma whipplei*, *Brucella*, *Legionella*, *Chlamydia*

## Abstract

The aim of this study was to investigate the prevalence of *Tropheryma whipplei, Chlamydia psittaci, Chlamydia pneumoniae, Legionella, Brucella,* and *Francisella tularensis* in valve samples from endocarditis patients using the real-time PCR method at a major referral heart hospital in Iran. In this study, 146 paraffin-embedded tissue samples from the heart valves of patients with clinical and pathological evidence of infective endocarditis (IE), who underwent heart valve replacement surgery between 2016 and 2020 at Tehran Heart Center were collected. After DNA extraction from paraffin-embedded valve tissue samples, they were surveyed for the presence of *T. whipplei, C. psittaci, C. pneumoniae, Legionella, Brucella,* and *F. tularensis* using quantitative real-time PCR (qPCR). The 16S rRNA gene sequence analysis was used for accurate species identification. Based on the molecular results, *T. whipplei*, *Chlamydia* spp., and *Legionella* spp. were detected in 10 (6.84%), 6 (4.1%), and 3 (2.05%) valve samples, respectively. In addition, one of six positive samples for *Chlamydia* spp. was identified as *C. psittaci*. No positive samples for *F. tularensis* and *Brucella* were found. In addition, all control valve samples were negative for all investigated pathogens. The findings suggest that specific bacterial species such as *T. whipplei*, *Chlamydia spp.,* and *Legionella spp*. are associated with the development of IE. Considering the life-threatening nature of IE, it is critical for healthcare systems to prioritize the identification of its causative agents and develop targeted treatment strategies.

**IMPORTANCE:**

Infective endocarditis (IE) is a serious and potentially life-threatening condition, and it is associated with significant morbidity, mortality, and complications, making it a major concern in both global and national healthcare systems. Late diagnosis and failure to receive appropriate treatment for patients with culture-negative endocarditis caused by hard-to-grow bacteria can lead to the death of patients. Unfortunately, in Iran, less attention is paid to the role of organisms that are difficult to cultivate in laboratory settings such as *Tropheryma whipplei, Chlamydia psittaci, Chlamydia pneumoniae, Legionella, Brucella,* and *Francisella tularensis* in causing culture-negative endocarditis, and these pathogens are overlooked by infectious, cardiologists, and health officials. This study underscores the need for special attention in the diagnosis of the agents of IE.

## INTRODUCTION

Infective endocarditis (IE) is a serious and potentially life-threatening condition characterized by inflammation of the inner lining of the heart chambers and valves, commonly caused by bacterial or fungal infections (1). It is associated with significant morbidity, mortality, and complications, making it a major concern in both global and national healthcare systems. The global burden of IE varies across different regions and populations. According to estimates from recent studies, the annual incidence of IE in the world ranges from 3 to 10 cases per 100,000 population (2). The prevalence of IE varies across different populations and is influenced by factors such as age, underlying cardiac conditions, healthcare access, and the presence of predisposing conditions such as intravenous drug use. The overall mortality rate associated with IE has been reported to range from 15% to 30%, with higher rates observed in specific subgroups such as elderly patients, those with prosthetic valves, and those with comorbidities. Mortality rates associated with IE can also be influenced by the causative organism (3, 4). Therefore, understanding the causative agents, epidemiology, prevalence, mortality rates, and associated complications of IE is crucial for effective management and resource allocation.

IE is primarily caused by microbial agents, with certain pathogens such as *Staphylococcus aureus, Streptococcus mutans, Streptococcus sanguinis,* and *Enterococcus* playing a more significant role than others (5). However, some rare bacterial species such as *Tropheryma, Chlamydia, Legionella, Brucella, and Francisella* are also associated with IE. The fastidious nature of these bacteria often prevents their growth on conventional culture media, posing significant challenges to their isolation and identification using traditional culture techniques (6). Alternative diagnostic methods, such as molecular testing, are essential to address the challenges associated with cases where blood cultures fail to detect the causative pathogens (6, 7).

Culture-negative endocarditis (CNE) is a form of IE where blood cultures fail to detect the causative pathogen, often due to prior antibiotic use, the slow growth of certain organisms, or their intracellular nature. Common agents of CNE include *Coxiella burnetii*, *Bartonella* species, *Tropheryma whipplei*, specific fungi, and atypical bacteria (8). In contrast, culture-positive endocarditis allows for the identification of pathogens like *S. aureus*, *Streptococci*, and *Enterococci* through standard blood culture, enabling targeted antibiotic therapy (8, 9).

Identifying CNE cases is essential for the accurate diagnosis and management of IE, as these cases often necessitate alternative diagnostic strategies. Fastidious organisms such as *Brucella* and *Francisella* present particular challenges, as blood culture sensitivity is limited to approximately 50%–70% (10). This limitation can lead to diagnostic difficulties, potentially delaying timely treatment and increasing the risk of complications. In contrast, molecular techniques like 16S PCR enable the direct detection of bacterial DNA, significantly improving the diagnostic yield in culture-negative cases (11, 12) and leading to improved management strategies for IE (13, 14). Due to this issue, the aim of this study was to conduct a comprehensive molecular investigation of *T. whipplei, C. psittaci, C. pneumoniae, Legionella, Brucella, and F. tularensis* in surgical valve samples obtained from patients diagnosed with culture-negative endocarditis at Tehran Heart Center valve samples.

## MATERIALS AND METHODS

### Study design and sampling

In this study, we examined medical histories and diagnostic reports of patients admitted to the Tehran Heart Center during 2016−2020 and underwent heart valve replacement. A total of 3,926 patient records and biopsy reports were reviewed. Additionally, the results of microbiological tests, including blood and valve tissue cultures of patients who exhibited pathological evidence of infection and clinical symptoms of endocarditis were examined. Of a total of 3,926 patient cases reviewed, we selected 146 cases based on strict inclusion criteria: each patient had undergone heart valve replacement surgery and exhibited both clinical and histopathological evidence consistent with IE (masses composed of fibrin, platelets, and infecting organisms, held together by agglutinating antibodies produced by the bacteria.). Only cases that met these criteria and showed no growth in blood or valve tissue cultures were included in the study, with confirmation from specialists. For the control group, 50 heart valve tissue samples were randomly selected from patients who underwent valve replacement during the same period for reasons unrelated to infection, with no clinical or pathological evidence of IE or any other infection. This selection process was designed to ensure that the control samples represented noninfectious cases accurately, thereby allowing for meaningful comparisons between infected and noninfected tissue.

The paraffin-embedded heart valve tissues were obtained from the pathology laboratory of Tehran Heart Center and transferred to the Epidemiology Laboratory at the Pasteur Institute of Iran. In the next step, the patient’s clinical files were reviewed, and their demographic information (e.g., sex, age, city, village of residence, contact with animals, immunosuppressive and underlying diseases, history of antibiotic use and its type, previous history of valve disease, clinical findings, as well as electrocardiography and echocardiography findings) were also recorded.

### DNA extraction

Several 5-µm pieces of tissue from each paraffin-embedded heart valve tissue were cut with a sterilized scalpel blade and then placed in sterilized microtubes. The chromosomal DNAs were extracted from deparaffinized heart valve tissue, using the GeneDiaTM FFPE DNA extraction Kit (Tehran, IRAN) according to the manufacturer’s instructions. The extracted DNA was kept at −20 °C until molecular testing. To ensure the quantity and quality of extracted DNA, spectrophotometric analysis was performed, measuring the DNA concentration and purity (A260/A280 ratio) to confirm optimal conditions for downstream applications. High-purity DNA with a ratio between 1.8 and 2.0 was considered acceptable for further testing.

The limit of detection (LOD) was experimentally determined by performing serial dilution of positive control DNA samples, identifying the minimum concentration detectable by qPCR for each pathogen. The LOD for *T. whipplei*, *Chlamydia spp*., *Legionella spp*., *Francisella spp., and Brucella spp.* is between 10^2^ and 10^3^, 10^1^ and 10^2^, 10^1^ and 10^3^, 10^1^ and 10^2^, and 10^2^ and 10^3^ copies per reaction in qPCR assays, respectively.

### Molecular diagnosis

Pathogen-specific real-time PCR assays were conducted for each targeted pathogen using either SYBR Green or TaqMan chemistry for sensitive and quantitative detection The PCR procedure was carried out by using the Corbett 6000 Rotor-Gene system (Corbett, Victoria, Australia), and 16S rRNA gene, was used as an internal control (15). Detailed conditions, primers, and protocols for each pathogen are provided in Table 1.

**TABLE 1:** Oligonucleotide sequences for qPCR amplification in this study

Bacteria	Gene	Primers	Probe	Size fragments (bp)
*F. tularensis*	ISFtu2	F:TTGGTAGATCAGTTGGTAGGATAACC R:TGAGTTTTATCCTCTGACAACAATATTTC	FAM-AAAATCCATGCTATGACTGATGCTTTAGGTAATCCA-TAMRA	97
*T. whipplei*	16S rRNA	Tws1F: TTGGTAGATCAGTTGGTAGGATAACC Tws1R: TGAGTTTTATCCTCTGACAACAATATTTC		203
*Brucella* spp.	IS711	F: GCTTGAAGCTTGCGGACAGT R: GGCCTACCGCTGCGAAT	FAM-AAGCCAACACCCGGCCATTATGGT-TAMRA	63
*Legionella* spp.	16S rRNA	F: CTAATTGGCTGATTGTCTTGAC R: CTGGCGATGACCTACTTTC		271
*Chlamydia spp.*	16S rRNA	F: CTTACAAGCCTTGCCTGTAGGG R: CCA AGTAGMGCAAGGATCGC		106

#### T. whipplei

The 16S rRNA gene was detected using a SYBR Green qPCR method, with a reaction mixture comprising AmpliQon Cyber Green Plus Master Mix (Ampliqon, Denmark). DNA from a clinical sample served as a positive control, whereas distilled water served as a negative control. The PCR cycling conditions included an initial activation at 95°C for 10 min, followed by 40 cycles of 95°C for 15 s, 59°C for 30 s, and 60°C for 60 s(16).

#### *Legionella* spp.

The 16S rRNA gene was detected using SYBR Green qPCR (Ampliqon, Denmark), with a positive clinical sample and distilled water as controls. PCR cycling conditions involved an initial activation at 95°C, followed by 40 cycles at 95°C for 15 s, 54°C for 45 s, and 59°C for 30 s (17).

#### *Chlamydia* spp.

Detection targeted the 16S rRNA gene using SYBR Green qPCR with (Ampliqon, Denmark) specified primers. Positive control (*L. pneumophila* NCTC 12007) and distilled water as negative control were employed, with cycling conditions of 95°C for 15 s, 57.5°C for 30 s, and 72°C for 30 s over 40 cycles (18).

#### F. tularensis

The TaqMan qPCR assay (AmpliQon, Denmark) targeted the ISFtu2 gene due to its high specificity, accurate identification capabilities, and low LOD, which enhances assay sensitivity. *F tularensis* subsp. *holarctica* was used as a positive control, whereas distilled water served as a negative control to ensure assay reliability (19).

#### *Brucella* spp.

The IS711 gene was targeted using a TaqMan-based qPCR assay due to its high specificity, ability to accurately identify pathogens, and lower LOD, which enhances assay sensitivity. Positive clinical samples and distilled water were included as controls to ensure precision and reliability (19).

### Sequence analysis and species confirmation

The positive samples were selected and sequenced at Genomein Company Tehran, Iran. The sequences were aligned manually and compared and analyzed with all sequences of the closely related species retrieved from GenBank database using jPhydit program version 1.1.3 (20). The relationship between our isolates and the standard established species was depicted by using a high bootstrap value phylogenetic tree of 16S rRNA gene by MEGA X software, using the neighbor-joining method with arithmetic mean of pairwise differences matrix.

### Statistical analysis

For qualitative variables, the mean, standard deviation, and median were calculated. Analytical analyses were performed to find the relationship between the presence of the gene and clinical and demographic findings. Logistic regression was used for univariate analysis. Variables with *P* values less than or equal to 0.15 were included in the model for multivariate analysis. All analyses were performed in SPSS version 23. Odds ratios and percentages were reported at 95% confidence intervals. *P*-values of less than 0.05 were considered statistically significant.

## RESULTS

### Demographic data

Of all surveyed patients included in the study, 28/91 (30.8%) patients and 7/53 (13.2%) patients were excluded from the study due to positive blood or valve tissue sample cultures, respectively. Ultimately, 146 non-duplicate culture-negative heart valves from 87 men and 59 women were examined. The mean age of male patients was 50.06, whereas that of female patients was 49.9, with a standard deviation of 14.25. A total of 14 patients (9.6%) succumbed to valve-related complications.

Regarding the valve distribution, 39.7% of the samples belonged to the aortic valve, 28.08% to the mitral valve, 17.8% to both valves (aortic and mitral), and other valves and heart tissue (tricuspid, aortic wall, and myocardium). Additionally, 28.7% of the patients had a history of valve replacement surgery, within them, 89.04% of the replaced with metal valves and 10.96% biological. In total, ≥20% of the patients had a fever (body temperature ≥38°C), and 98% had a history of vancomycin use.

Eight women reported a history of abortion, but samples from these women did not reveal the presence of *Coxiella* or *Bartonella*. Based on pathological findings, subclinical endocarditis was diagnosed in 21.2% of the patients, and vegetation legion was reported in 8.2% of the patients. Furthermore, more than 70% of the patients showed heart tissue inflammation based on pathological findings. Table 2 provides detailed demographic data on the patients with positive qPCR results.

**TABLE 2:** Demographic data of patients with positive qPCR results

Bacteria	ID	Gender (F/ M)	Age	Clinical symptoms							Surgical valve type	Underlying condition or other clinical representation
				Fever	Limb edema	Shortness of breath	Cough	Weakness	Hypertension	Loss weight		
*T. whipplei* (*N* = 10, 6.84%))	E-121	F	36	No	No	Yes	No	No	Yes	No	AVR^*a*^/MVR^*b*^	No
	E-79	F	68	Yes	No	Yes	No	No	No	No	MVR ,CABG^*c*^	Tachycardia, chest pain, history of miscarriage, and cardiovascular diseases
	E-66	M	72	Yes	No	Yes	Yes	Yes	Yes	No	CABG, AVR	Metal valve, died
	E-71	F	40	No	No	Yes	No	No	No	No	MVR	History of Mitral valve replacement
	E-48	F	53	Yes	No	Yes	No	No	No	No	MVR and CABG	History of heart and kidney disease and miscarriage
	E-75	F	63	Yes	No	Yes	Yes	Yes	No	Yes	AVR	History of CABG in April 2016, diabetes and spleen surgery
	E-108	M	64	Yes	No	Yes	No	No	Yes	No	TVR^*d*^, Redo MVR	History of Mitral valve replacement
	E-96A	M	40	Yes	No	Yes	No	No	No	Yes	MVR and AVR	History of acute chronic IE
	E-89	F	55	Yes	No	Yes	No	No	Yes	No	MVR and AVR	History of cardiovascular disease and angiography, kidney problems and abortions
	E-38	F	53	Yes	No	Yes	No	No	No	No	MVR CABG	History of cardiovascular disease, kidney problem and abortion, History of acute IE
*Legionella* sp. (*N* = 3, 2.05%)	E-215	M	37	Yes	Yes	Yes	Yes	Yes	No	Yes	AVR	Skin rash, fatigue and sweating, History of IE
	E-127	M	78	Yes	No	Yes	No	Yes	Yes	Yes	redo MVR and redo AVR	History of aortic and mitral valve replacement, biological type valve, history of IE, died
	E-109	M	34	Yes	No	Yes	No	Yes	No	No	redo MVR	History of aortic and mitral valve replacement
*Chlamydia* sp. (*N* = 6, 4.1%)	E-59A	M	27	Yes	Yes	Yes	No	No	No	No	AVR,MVR	Splenomegaly, History of IE
	E-52	M	54	No	No	Yes	Yes	No	No	No	MVR	History of IE and kidney disease
	E-124	M	68	Yes	Yes	Yes	Yes	Yes	Yes	No	redo MVR	History of mitral valve replacement, biological type valve, History of IE with *Coxiella burnetii*
	E-111	M	37	Yes	No	Yes	No	Yes	No	Yes	AVR	History of angiography and IE
	E-220	M	33	Yes	Yes	Yes	Yes	Yes	No	Yes	AVR/MVR	No
	E-55B	F	77	Yes	No	Yes	No	Yes	No	No	AVR	Kidney disease, died

^*a*^ Aortic valve replacement.

^*b*^ Mitral valve replacement.

^*c*^ Coronary artery bypass graft.

^*d*^ Transcatheter valve replacement.

### Pathogen identification

We have not detected any positive samples among the control group. Additionally, of the 146 heart valve samples from patients with culture-negative endocarditis, none of the samples was found to be infected with *F. tularensis* and *Brucella* sp.

A total of 10 patients (6.84%) tested positive for *T. whipplei* using qPCR, followed by 6 patients (4.1%) for *Chlamydia* sp., and 3 patients (2.05%) for *Legionella* sp. (Table 2).

### Sequencing and phylogenetic analysis

All 19 positive qPCR products were sequenced using specific primers for 16SrRNA gene.

**Fig 1:**
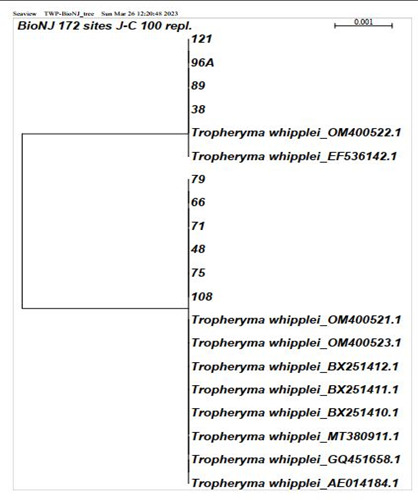
Phylogenetic tree of positive samples of *T. whipplei* depicted by SeaView version 4 software and the neighbor-joining algorithm with bootstrap 100.

The partial 16SrRNA gene sequences analysis of isolates showed that our isolates had high similarity (100%) to the nearest validated species. The isolates E-121, E-79, E-66, E-71, E-48, E-75, E-108, E-96A, E-89, and E-38 belonged to *T. whipplei* species, and the isolates E-59A, E-52, E-124, E-111, E-220, and E-55B belonged to genus *Chlamydia* sp. The sequencing results revealed that sample E-52 belonged to the *C. psittaci* species. The data analysis involved the classification of isolates E-215, E-127, and E-109 within the *Legionella* genus, without further discrimination at the species level. The relationship between our isolates and validated established species was depicted using a high bootstrap value phylogenetic tree of the 16SrRNA gene by SeaView version 4 software. The neighbor-joining method with an arithmetic mean of pairwise differences matrix was used (Fig. 1).

## DISCUSSION

Endocarditis involves the development of vegetation on cardiac valves. It can occur as a result of direct infection of the valve structure, typically occurs in patients with pre-existing valve damage who have bacteremia (21), or as a chronic, low-grade thrombotic event in patients with acquired thrombophilia related to conditions such as cancer, sepsis, or autoimmune disorders (known as Non-Infective Endocarditis [NIE]). Although distinguishing between IE and NIE at the macroscopic level is challenging, these two forms of endocarditis exhibit distinct clinical features and can be readily differentiated through microscopic pathology (22, 23). A cohort study found that of the 814 cases of endocarditis, 409 were classified as IE, and 405 classified as NIE (24).

From a clinical perspective, IE is more commonly diagnosed than NIE (24). Conversely, patients with IE often experience recurrent fever, prompting them to seek medical attention. On the other hand, NIE is typically diagnosed incidentally during imaging tests performed for other reasons or post-mortem examinations. Several case series based on post-mortem findings have reported the presence of NIE in patients with various clinical conditions, predominantly cancer-related cases (25).

IE is a major cause of death, with an annual prevalence of 1.5–15 cases per 100,000 people per year (26). IE remains a major medical concern due to its high mortality rate, which can be fatal if not properly treated. Epidemiological studies show that the prevalence of IE is increasing (27). On the other hand, reports of the prevalence of negative blood culture endocarditis vary significantly in different regions of the world (13%–69%) (28). Fast-growing and non-cultivable organisms account for approximately half of all blood culture-negative endocarditis worldwide (10) Currently, valve replacement surgery is required in 20%–40% of patients with IE, but the culture of heart valve tissue samples is generally unreliable for diagnosing the causative agent of endocarditis (5). The present study investigated the presence of various infectious agents in the replaced valves of 146 patients who had culture-negative endocarditis and underwent valve replacement surgery. Among the patients, *T. whipplei* infection was detected in 10 cases, representing a prevalence rate of 6.84%. *T. whipplei* is a bacterium known to cause Whipple’s disease, a rare systemic infectious disorder. The detection of this bacterium in a significant proportion of patients with culture-negative endocarditis highlights its potential involvement in this condition. Furthermore, *Chlamydia* infection was detected in the replaced valves of six patients, accounting for a prevalence rate of 4.1%. Subsequent investigations revealed that one of these cases was positive for *C. psittaci*.

Chlamydial infections have been implicated in various clinical manifestations, including endocarditis, and the identification of *C. psittaci* emphasizes its potential role in this context (29). In addition, *Legionella* infection was observed in the valves of three patients (2.05%) who had endocarditis with negative cultures. *Legionella* species are known to cause Legionnaires’ disease, a severe form of pneumonia. The identification of *Legionella* infection in these cases suggests its possible contribution to culture-negative endocarditis. However, no positive samples for *F. tularensis*, the causative agent of tularemia, or *Brucella* bacteria were found among the examined samples of patients.

Our findings suggest that the prevalence of the studied pathogens in Iran might be relatively low, which could explain why they may not significantly contribute to the development of culture-negative endocarditis within our study cohort. This observation aligns with previous research indicating regional variations in the etiology of infectious diseases, including endocarditis (30, 31). Geographical location, climate, population demographics, and healthcare practices are key determinants that influence the distribution and prevalence of infectious agents associated with endocarditis.

Therefore, our study underscores the importance of considering regional epidemiological patterns when assessing the etiology of culture-negative endocarditis and designing appropriate diagnostic and treatment strategies tailored to specific geographic regions.

The findings of this study shed light on the infectious etiology of culture-negative endocarditis, providing insights into the prevalence of specific pathogens. The identification of *T. whipplei*, *Chlamydia* (including *C. psittaci*), and *Legionella* as potential causative agents expands our understanding of the microbial spectrum associated with this condition. In an observational cohort study by Geißdörfer et al, Whipple’s tropheryma was detected in 16 of 1,135 patients whose heart valves were examined (32). Also, Kestler and colleagues investigated that anaerobic bacteria caused 22 cases of IE, and *Propionibacterium acnes*, *Lactobacillus* species, and *Clostridium* species were the most common pathogens (33). Finally, in a preliminary study of cerebral embolization, Cooper and colleagues in America identified infection with *S. aureus* as an important risk factor for embolization in patients with left-sided IE (34).

The use of molecular tests allowed for the identification of specific pathogens in these cases. Further research is necessary to explore the clinical implications, optimal diagnostic strategies, and appropriate management approaches for these infections in the context of culture-negative endocarditis. Lamas and colleagues reported 1 case of *Chlamydia* in 15 patients with culture-negative IE during the study period (35). *T. whipplei* was diagnosed in 3.5% of IE patients in northern and central Europe, which is consistent with the present study (36, 37). Therefore, it can be said that bacterial infections in the heart valve are a primary cause of endocarditis. In addition, due to the spread of *T. Whipplei*, *Legionella*, and *Chlamydia* in culture-negative endocarditis patients, these causative agents should also be considered in further studies. Molecular genetic screening for bacteria is a very useful diagnostic strategy, especially in cases of heart valve replacement. In a study by Pearce and colleagues, they reported a case of endocarditis caused by *Legionella* using the qPCR method (38). In another study, Massey and colleagues identified a case of endocarditis caused by *Legionella* (39). Littrup and colleagues also identified a case of *Legionella* endocarditis in a 38-year-old hospitalized woman by molecular method (40). However, Rovery and colleagues conducted a study on PCR detection of bacteria on the heart valves of patients with bacterial endocarditis under treatment. They reported that PCR results should be interpreted with caution in a patient with a history of previously treated IE, as bacterial DNA can persist in the heart valve long after the resolution of IE (9).

In this study, among the 19 valve samples that tested positive for bacterial DNA by PCR, a correlation was observed between molecular findings and histopathological signs of IE. Specifically, 12 of these samples (63%) showed classic histopathological indicators of IE, including inflammation, tissue necrosis, and fibrinous vegetations. For instance, samples positive for *T. whipplei* were associated with moderate inflammation and focal necrosis, suggesting an active infection consistent with the presence of bacterial DNA. Similarly, in the cases where *Chlamydia spp.* was detected, histopathological examination revealed fibrin deposits and mild to moderate inflammatory infiltrates, further supporting the molecular findings. However, in seven samples (37%), PCR-positive results did not correlate with significant histopathological changes, showing only minimal or no visible signs of inflammation. These cases, particularly among samples positive for *Legionella spp.*, exhibited low-level bacterial DNA but lacked substantial inflammatory or necrotic markers in tissue, which could indicate either a latent infection or transient bacterial presence without active involvement in IE pathology. These findings underscore the value of integrating PCR results with histopathological evidence to more accurately interpret the clinical relevance of detected pathogens. Cases with both molecular and histological confirmation support an active role of the pathogen in IE, whereas discrepancies, such as the presence of microbial DNA without histological damage, highlight the need for careful interpretation of PCR results in diagnosing IE.

### Limitation of study

The limitations of this study include the lack of a comprehensive histopathological correlation for all PCR-positive cases, which restricts the ability to confirm whether the detected bacterial DNA represents active infection or latent presence without tissue damage. Resource constraints also limited PCR testing to culture-negative cases, excluding culture-positive samples that could have provided valuable comparative insights. Additionally, the single-center design and relatively small sample size may impact the generalizability and statistical robustness of the findings. Finally, the absence of longitudinal follow-up data prevents the assessment of patient outcomes in relation to PCR-positive results, limiting insight into the clinical implications and treatment efficacy for culture-negative IE. These limitations underscore the need for future studies to incorporate histopathological assessments, a broader, multi-center sample, and follow-up data to better understand the role of molecular diagnostics in IE.

### Conclusion

In conclusion, late diagnosis and failure to receive appropriate treatment for patients with culture-negative endocarditis caused by hard-to-grow bacteria can lead to the death of patients. Unfortunately, in Iran, less attention is paid to the role of organisms that are difficult to cultivate in laboratory settings such as *F. tularensis*, *T. whipplei*, *Brucella*, *Legionella*, *C. psittaci*, and *C. pneumoniae* in causing culture-negative endocarditis, and these pathogens are overlooked by infectious, cardiologists, and health officials. This study underscores the need for special attention in the diagnosis of the agents of IE.
